# Anti-Inflammatory Drug Therapy in Aneurysmal Subarachnoid Hemorrhage: A Systematic Review and Meta-Analysis of Prospective Randomized and Placebo-Controlled Trials

**DOI:** 10.3390/jcm12124165

**Published:** 2023-06-20

**Authors:** Johannes Wach, Martin Vychopen, Agi Güresir, Erdem Güresir

**Affiliations:** Department of Neurosurgery, University Hospital Leipzig, 04103 Leipzig, Germany; martin.vychopen@medizin.uni-leipzig.de (M.V.); agi.gueresir@medizin.uni-leipzig.de (A.G.); erdem.gueresir@medizin.uni-leipzig.de (E.G.)

**Keywords:** intracranial aneurysm, treatment, subarachnoid hemorrhage, vasospasm, delayed ischemic neurological deficit

## Abstract

Emerging evidence suggests that neuroinflammation may play a potential role in aneurysmal subarachnoid hemorrhage (aSAH). We aim to analyze the influence of anti-inflammatory therapy on survival and outcome in aSAH. Eligible randomized placebo-controlled prospective trials (RCTs) were searched in PubMed until March 2023. After screening the available studies for inclusion and exclusion criteria, we strictly extracted the main outcome measures. Dichotomous data were determined and extracted by odds ratio (OR) with 95% confidence intervals (CIs). Neurological outcome was graded using the modified Rankin Scale (mRS). We created funnel plots to analyze publication bias. From 967 articles identified during the initial screening, we included 14 RCTs in our meta-analysis. Our results illustrate that anti-inflammatory therapy yields an equivalent probability of survival compared to placebo or conventional management (OR: 0.81, 95% CI: 0.55–1.19, *p* = 0.28). Generally, anti-inflammatory therapy trended to be associated with a better neurologic outcome (mRS ≤ 2) compared to placebo or conventional treatment (OR: 1.48, 95% CI: 0.95–2.32, *p* = 0.08). Our meta-analysis showed no increased mortality form anti-inflammatory therapy. Anti-inflammatory therapy in aSAH patients tends to improve the neurological outcome. However, multicenter, rigorous, designed, prospective randomized studies are still needed to investigate the effect of fighting inflammation in improving neurological functioning after aSAH.

## 1. Introduction

Aneurysmal subarachnoid hemorrhage (aSAH) is a severe neurological disorder that affects 6–9 individuals per 100,000 each year and has a mortality rate of 35% [1]. In recent decades, the incidences of aSAH have decreased globally due to reduced smoking rates and improved management of arterial hypertension [2]. However, mortality and morbidity rates remain high, and survivors often experience poor functional outcomes, with roughly half unable to return to their previous life [3]. Prehospital mortality rates from aSAH are still between 22% and 26% [4]. Additionally, about 35% of aSAH patients suffer from memory disturbances, depression, and reduced quality of life [5,6,7,8].

Cerebral vasospasm (CVS) is linked to delayed cerebral ischemia (DCI) and is believed to play a role in unfavorable results following aSAH [9]. In order to enhance outcomes, various rescue therapies are utilized, including the selective intra-arterial infusion of vasodilators, balloon angioplasty, and induced hypertension [3,10]. However, the underlying pathophysiological mechanisms that enable injury expansion following aSAH are still not fully understood, and only a limited number of pharmacological treatments have been proven effective. Recent data suggest that neuroinflammation has a pivotal effect in injury expansion and neurological deficits [11,12,13]. Immune cells from the periphery are recruited into the brain parenchyma, and their activated forms release inflammatory cytokines [14,15]. Vessels affected by cerebral vasospasm have been found to have an elevated leukocyte adhesion capacity, resulting in delayed neurologic deterioration [16,17].

Numerous previous clinical trials have investigated the use of various anti-inflammatory drugs in aSAH patients to improve mortality and functional outcomes. In this meta-analysis, we aim to examine the available evidence and identify effective drug interventions that could improve functional outcomes and survival rates in aSAH patients.

## 2. Materials and Methods

To carry out this systematic review, we utilized the Cochrane Collaboration format [18] and adhered to the PRISMA checklist (see Supplementary Figure S1) [19]. The study was registered in the “*International Prospective Register of Systematic Reviews*” (PROSPERO) in 2023 (CRD42023395375), and a comprehensive, predetermined protocol can be obtained upon request.

### 2.1. Search Strategy for Identification of Studies

The authors performed a systematic search of the Pubmed database (http://www.ncbi.nlm.nih.gov/pubmed) using the terms “aneurysmal subarachnoid hemorrhage”, “SAH”, and “subarachnoid hemorrhage”. The search was limited to randomized controlled trials, human studies, and English-language articles. The authors screened the articles for randomized placebo, controlled trials investigating anti-inflammatory drug therapies. The literature search encompassed all results until 31 March 2023. The inclusion criteria were developed based on the PICOS (population, intervention, comparator, outcomes, and study design) framework [20]. The following criteria were applied: patients suffered from aSAH; anti-inflammatory drug therapies were implemented; results were compared to a placebo arm; all results of the prespecified endpoints were reported; and the trials were defined as prospective, randomized, placebo-controlled, and double-blinded. The authors excluded certain types of results, such as reviews, letters, study protocols, conference abstracts, unpublished manuscripts, animal experiments, and studies with insufficient data (e.g., randomized controlled trials without a placebo arm). Furthermore, we conducted a search for studies that matched our inclusion and exclusion criteria in previous meta-analyses and systematic reviews. The identified articles underwent a stepwise evaluation process: Initial screening of study titles, followed by assessment of corresponding abstracts, and in case of uncertainty, the full-text screening by two authors (JW and EG) until all retrieved studies were either included or excluded.

### 2.2. Types of Studies

Randomized, double-blind, placebo-controlled clinical trials that evaluated the efficacy of anti-inflammatory treatment in improving the outcome and survival rates of aneurysmal subarachnoid hemorrhage were included. Trials without survival or neurological outcome data were excluded. Trials that specifically investigated elderly or geriatric aSAH patients (>60 years) were excluded. Neurological outcome was measured using the modified Rankin Scale (mRS). If an article presented numerical data for individual mRS classes, the data were documented based on the definition of a poor outcome as a score ranging from 3 to 6 on the mRS scale, while a score ≤ 2 was considered indicative of a good outcome [21]. If a study presented the percentage of patients with a defined outcome event, the absolute numbers were computed using the provided percentages.

### 2.3. Data Extraction and Quality Evaluation

Baseline data extracted from the studies included the study names, first authors, publication year, country, number of centers (whether mono-, bi-, or multicentric), key trial design characteristics (such as randomization method, allocation method, presence of missing data, lack of reported outcome, and utilization of intention-to-treat analysis), as well as other pertinent information.

### 2.4. Statistics

The meta-analyses were performed using Review Manger Web (RevMan Web Version 5.4.1 from The Cochrane Collaboration). Statistical heterogeneity was assessed using the x^2^, while inconsistency was evaluated using the I^2^ statistics. Substantial heterogeneity was considered when the I^2^ value reached 50% or higher. The weight of each individual trial’s contribution to the treatment effect estimation was determined based on the study sample size. Data from a multi-arm trial that involved different dosage regimens were combined to create a unified group for inclusion in the pairwise meta-analysis [22]. To assess publication bias for the defined endpoints of survival and neurological outcome, three methods were applied. Firstly, funnel plots were generated to visually examine the publication bias of the included trials. Secondly, an Egger regression analysis was conducted to statistically evaluate the symmetry of the funnel plot. The likelihood of publication bias was determined using the two-tailed Egger regression intercept test, with a significance threshold set at 5% [23]. Thirdly, Begg’s test was utilized to evaluate the symmetry of the data [24]. MedCalc (Version 20.123 for Windows) was used to perform both Egger’s and Begg’s tests. Pooled odds ratio (OR) estimates, using a random effects model, were used to express the effect sizes. Analyses were conducted for both death and poor outcome.

## 3. Results

### 3.1. Literature Search

**Figure 1:**
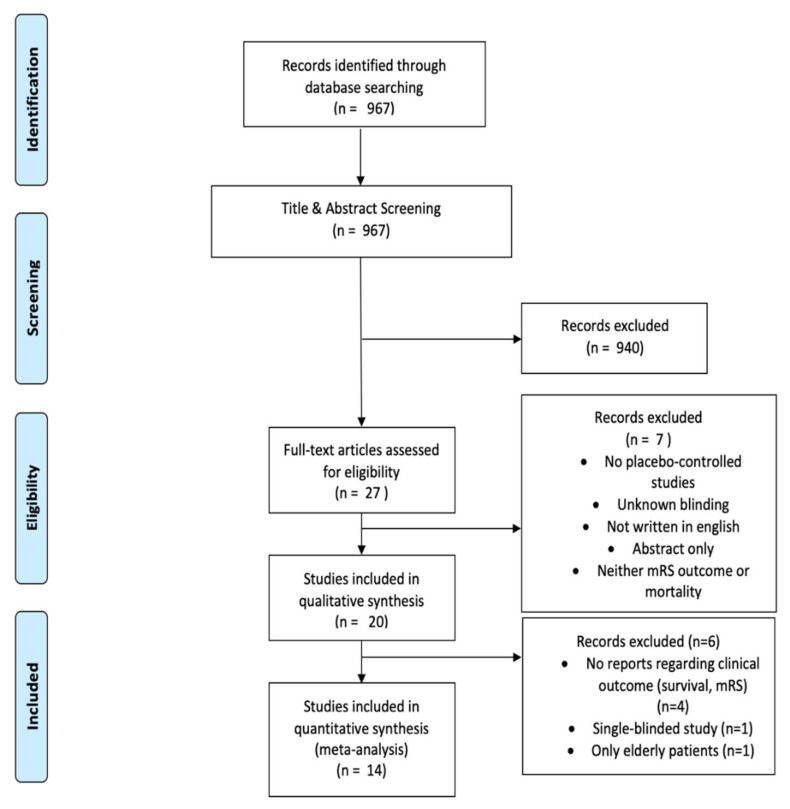
PRISMA flow chart showing the study selection of the present meta-analysis.

Based on the search strategy, a total of 967 English articles (Figure 1) were deemed eligible. Following the evaluation of the titles, abstracts, and full texts, 953 articles were excluded. Ultimately, 14 articles encompassing 1759 patients were included for the meta-analysis.

### 3.2. Characteristics of Included Studies

The included studies were published from 1989 to 2022. Table 1 shows the major characteristics of the anti-inflammatory drug interventions of the 14 included trials [25,26,27,28,29,30,31,32,33,34,35,36,37,38]. The participants included in each study were all aneurysmal SAH patients. The duration of treatments varied from 24 h to 21 days. One prospective, double-blind, and placebo-controlled trial was designed as a multi-arm trial [34]. Data were included by combining groups into the pairwise meta-analysis of our dichotomous endpoints. Further identified anti-inflammatory prospective studies in aSAH that did not fulfill our inclusion criteria due to single-blinded design, unknown blinding method, investigating elderly patients only, written in other languages than English, unavailable data for the primary endpoints of this meta-analysis, or the lack of placebo arms are provided in the Supplementary Table S1 [39,40,41,42,43,44,45,46,47,48,49,50,51].

### 3.3. Risk of Bias and Quality Assessment

**Figure 2:**
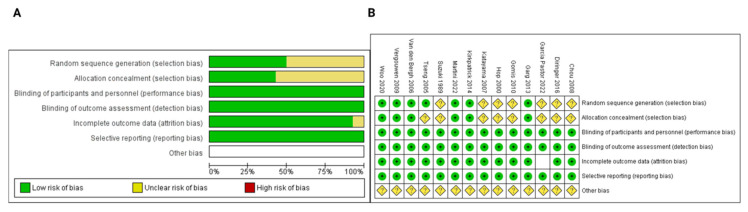
(**A**) Risk of bias evaluation for each kind of bias. (**B**) Summary of risk of bias of the individual randomized controlled trials (reviewers’ judgments about each risk of bias characteristic of the included studies: “+” constitutes low risk; “?” constitutes unclear risk).

All included trials used a double-blinded design to minimize performance bias and detection bias of the personnel and patients. Furthermore, all trials were defined as randomized trials. The randomization technique was not reported in seven trials [25,26,27,29,30,31,34]. All prespecified endpoints are reported with the corresponding outcomes. Generally, no prospective, randomized, double-blind, and placebo-controlled trial showed characteristics that might indicate a high risk of bias. The frequency of the individual bias of each trial and the overall bias assessment are displayed in Figure 2. The detailed quality assessment protocol regarding risk of bias and author judgments for the individual studies are given in Supplementary Table S2.

### 3.4. Impact of Anti-Inflammatory Therapy in Aneurysmal SAH on Survival

**Figure 3:**
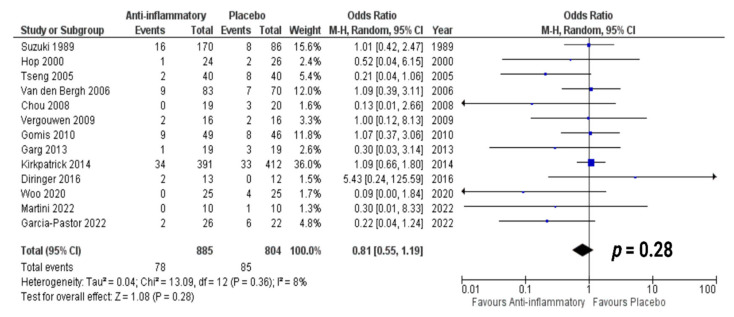
Forest Plots displaying OR and 95% CI estimates for death in studies evaluating anti-inflammatory therapies compared to conventional therapy in aSAH [25,26,27,28,29,30,32,33,34,35,36,37,38]. Squares constitute the odds ratio; the bigger the square, the greater the weight given because of the narrower 95% CI. Diamond shows the odds ratio of the overall data.

A total of 13 studies met our selection criteria and reported data regarding survival [25,26,27,28,29,30,32,33,34,35,36,37,38]. A total of 1689 patients were randomized into either anti-inflammatory (*n* = 885) or placebo treatment (*n* = 804). A total of 78 patients (8.8%) out of 885 in the anti-inflammatory treatment arm died, and 85 (10.6%) of the 804 patients in the control arms died. The outcome evaluation ranged from one month to one year after treatment. The overall odds ratio (Figure 3) for death in the pooled analysis was 0.81 (95% CI: 0.55–1.19) (*p* = 0.28). No significant heterogeneity was present (I^2^ = 8%, *p* = 0.36). No studies were identified that indicated a significant positive or negative impact of anti-inflammatory therapy on the outcome endpoint of “death”.

### 3.5. Impact of Anti-Inflammatory Therapy in Aneurysmal SAH on Neurological Outcome

**Figure 4:**
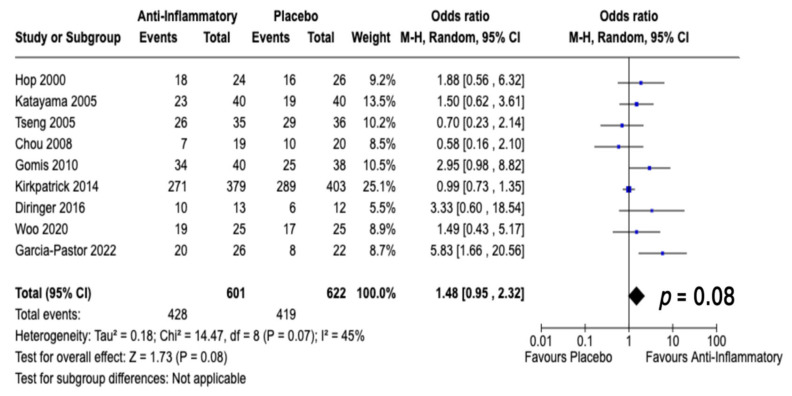
Forest Plots displaying OR and 95% CI estimates for a good neurological outcome (using mRS grading: mRS ≤ 2) in studies evaluating anti-inflammatory therapies compared to placebo therapy in aneurysmal SAH [25,26,27,29,30,31,32,35,38]. Squares constitute the odds ratio; the bigger the square, the greater the weight given because of the narrower 95% CI. Diamond shows the odds ratio of the overall data.

A total of 9 studies met our selection criteria and reported data regarding neurological outcome, which enabled a dichotomization into mRS ≤ 2 or mRS > 2 [25,26,27,29,30,31,32,35,38]. A total of 1223 patients were randomized into either anti-inflammatory (*n* = 601) or control treatment (*n* = 622). In total, 428 patients (71.2%) out of 601 in the anti-inflammatory treatment arm achieved a good neurological outcome (mRS ≤ 2), and 419 (67.4%) of the 622 patients in the placebo arms achieved a favorable outcome. The outcome evaluation ranged from one month to one year after treatment. The overall odds ratio (Figure 4) for mRS ≤ 2 in the pooled analysis was 1.48 (95% CI: 0.95–2.32) (*p* = 0.08). No significant heterogeneity was present (I^2^ = 45%, *p* = 0.07). One study investigating the effect of dapsone reported a beneficial effect regarding functional outcome as endpoint [27]. Furthermore, cerebrolysin also resulted in an increased probability to achieve a favorable outcome compared to placebo treatment [38].

### 3.6. Publication Bias

**Figure 5:**
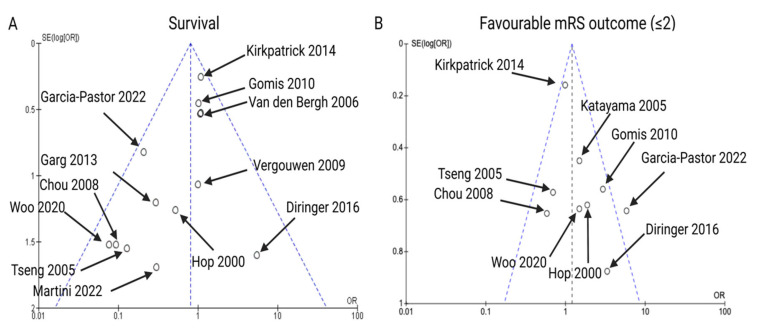
(**A**) Funnel plot visualizing the analysis of publications bias for the endpoint survival in 13 studies [25,26,27,28,29,30,32,33,34,35,36,37,38]. (**B**) Funnel plot visualizing the analysis of publications bias for the endpoint neurological outcome in nine studies [25,26,27,29,30,31,32,35,38].

In order to ensure a reliable assessment, several measures were implemented to examine the presence of publication bias. Firstly, a comprehensive literature search strategy was applied. Secondly, the selected studies included in this meta-analysis strictly adhered to the predefined inclusion and exclusion criteria. Thirdly, publication bias was evaluated using funnel plots (see Figure 5) for the endpoints (survival and favorable mRS outcome (≤2)). Finally, statistical analyses of publication bias using Egger´s and Begg´s tests were performed. The data points for survival analysis were all positioned within the inverted funnel, indicating no visually apparent significant publication bias in relation to the survival analysis.

Following the visual examination for publication bias, both Egger’s and Begg’s tests were conducted to investigate the presence of publication bias for the primary outcomes. Regarding mortality, Egger´s test indicated a statistically significant publication bias (*p* = 0.03, intercept = −1.05, 95% CI −1.99–−0.11), while Begg´s test revealed a Kendall’s tau of −0.23 (*p* = 0.27). We identified one study investigating dapsone in aSAH with a very high effect size in the bottom-right corner of the funnel plot regarding favorable mRS outcome analysis [26]. Hence, there might be the risk that there is an underbelly of unpublished studies with similar standard errors, but small and non-significant effects. Furthermore, Egger´s test did not indicate any significant publication bias concerning the endpoint of “favourable mRS outcome (≤2)” (*p* = 0.10, intercept = 1.23, 95% CI −0.30–2.76), and Begg’s test yielded a Kendall’s tau of 0.29 (*p* = 0.25).

## 4. Discussion

The present meta-analysis summarized available data on anti-inflammatory therapy in aSAH and suggests a potential need for further trials investigating anti-inflammatory therapies using dapsone or corticosteroids. These drugs may positively influence the neurological outcome after aSAH. Our results indicate that anti-inflammatory therapy may have a positive effect on functional outcome. This provides valuable evidence for clinicians when considering the prescription of effective medications for patients with aSAH. Nimodipine remains the benchmark medical drug therapy for preventing DCI and influencing the outcome in aSAH patients [3,52]. In the following section, we will discuss the findings of the present meta-analysis and debate the classes of investigated anti-inflammatory drugs.

### 4.1. COX Inhibition

Cyclooxygenase (COX) is a bifunctional enzyme that acts as a cyclo-oxygenase and catalase. The conversion of arachidonic acid to prostaglandins is facilitated by the enzymes COX-1 and COX-2. COX-1 synthesizes thromboxane A2, which induces platelet aggregation, vasoconstriction, and proliferation of smooth muscles. In contrast, COX-2 synthesizes prostacyclin, which induces vasodilation and relaxation of smooth muscles and antagonizes thromboxane A2 in the macrovascular endothelium [53,54]. Animal investigations have shown that arterial endothelial cells have an increased expression of COX-2 after SAH, while the expression of COX-1 remains unchanged [55]. Animal trials have shown that inhibiting COX-2 may prevent brain edema and preserve neurological functioning [56]. However, non-selective COX-2 inhibition by nonsteroidal anti-inflammatory medications, such as acetylsalicylic acid, might elevate the risk of re-bleeding, despite attenuating neuroinflammation in aSAH [57,58]. Although acetylsalicylic acid failed to significantly reduce the risk of a delayed ischemic neurological deficit and improve the functional outcome in prospective, double-blind, and placebo-controlled trials [30,36], there was a tendency to prescribe it, as its relative risk reduction for poor outcome was 21% [36]. Celecoxib, a selective COX-2 inhibitor, has not been prospectively investigated in randomized and double-blinded aSAH trials yet. A small clinical trial on subjects with intracerebral hemorrhage showed that celecoxib reduced hematoma and edema volume through its anti-inflammatory effect [59]. However, the current limited evidence for celecoxib therapy in aSAH is due to the failure of acetylsalicylic acid and the known increased risk of myocardial infarction with celecoxib therapy [60].

### 4.2. Thromboxane A2 Synthetase Inhibition

Thromboxane A2 (TXA_2_) is a prothrombotic prostanoid that is predominantly synthesized via COX-1, COX-2, and TXA_2_ synthase [61]. Furthermore, it is released by activated platelets in the case of inflammation or tissue injury [62]. Afterwards, TXA_2_ binds to the TXA_2_ receptor, resulting in smooth muscle cell constriction and platelet aggregation. Experimental SAH rat models showed that the SAH-induced regional blood flow reduction is closely linked to an increased expression of TXA_2_ receptors in the smooth muscle cells of cerebral arteries and microvessels [63]. An increase of TXA_2_ levels induces leukocyte aggregation and systemic inflammation. However, the already debated acetylsalicylic acid can reduce the TXA_2_ levels by inactivating COX-1 [64], whereas nimodipine exerts no effect on serum thromboxane levels [65]. The prospective randomized double-blind trial of Suzuki et al. [34] showed a potential use of a thromboxane synthetase inhibitor in preventing vasospasm and infarcts in severe aSAH cases with Hunt & Hess grades III and IV. However, the results of this trial regarding functional outcome could not be included in the statistical analysis because they were not transferrable to the mRS scale.

### 4.3. Epoxide Hydrolase Inhibition

Soluble epoxide hydrolase is the metabolizing enzyme of epoxyeicosatrienoic acids, which oppose the expression of vascular cell adhesion molecule-1 (VCAM-1) by blocking the NF-κB translocation. The translocation NF-κB is an important part of the expression of the pro-inflammatory adhesion molecule VCAM-1 [66,67]. The consequence of this vascular inflammatory pathway is the development of a vasogenic edema, causing the extravasation of ions and proteins through a disturbed blood–brain barrier [68]. Animal experiments have shown that the genetic deletion or inhibition of soluble epoxide hydrolase results in a reduced expression of VCAM-1 in the cerebrovasculature, thereby attenuating the transmigration of infiltrating inflammatory cells [69,70]. Increased levels of VCAM-1 have been observed in the plasma or CSF of aSAH patients, and this inflammatory response is suggested to be an important part of vasogenic edema development [69,71]. The first human prospective, randomized, and placebo-controlled trial investigating soluble epoxide hydrolase inhibition was recently conducted by Martini et al. [33], which showed that the application is safe for humans, and there was no drug-associated mortality. Furthermore, it was revealed that soluble epoxide hydrolase inhibition resulted in increased serum levels of epoxyeicosatrienoic acids, whereas the cerebrospinal fluid levels of epoxyeicosatrienoic acids were not sufficiently increased by the application of the soluble epoxide hydrolase inhibitor. To date, the current evidence is limited by the low power size, and the results of the present meta-analysis cannot recommend a routine clinical application. Further larger trials of soluble epoxide hydrolase inhibitors with an improved CNS penetration might also provide information regarding the functional outcome.

### 4.4. Statins

Statins, which are commonly referred to as HMG-CoA reductase inhibitors, have pleiotropic effects, including anti-inflammatory actions and the attenuation of vasospasm. Statins enhance the expression and activity of endothelial nitric oxide synthase and reduce nitric oxide scavenging by free radicals [72,73]. Moreover, they can inhibit the activation and migration of inflammatory perivascular granulocytes [74,75], potentially attenuating or preventing cerebral vasospasms after aSAH. To date, eight randomized [25,26,28,35,37,44,45,46] and four large multicenter, prospective, randomized trials [32,43,49,51] have evaluated the use of simvastatin, atorvastatin, pitavastatin, or pravastatin in aSAH patients. However, 2 studies had a special focus on either elderly patients over 60 years [39] or a dose comparison of simvastatin without a placebo group [51]. The primary endpoints of these studies were the frequency of vasospasm, presence of DCI, and the functional outcome. A previous meta-analysis, which summarized the results of some of the aforementioned trials focused on the potential reduction of vasospasm (defined by transcranial doppler sonography), found no significant effect [76]. However, another meta-analysis, which exclusively focused on statins and the treatment duration, found that a statin therapy for 2 weeks may improve neurological outcome. Surprisingly, prolonged use for 3 weeks had no significant effect on neurological outcome [77]. In the present meta-analysis, we also could not observe a significant effect on mortality or functional outcome. Current evidence and guidelines do not recommend routine use of statins in aSAH [3,77,78]. To assess the impact of statin treatment over a duration of two weeks, it is necessary to conduct future large-scale trials. The recently published AHA/ASA 2023 aSAH guideline does not recommend the routine use of statin therapy regarding the prevention of delayed cerebral ischemia [4].

### 4.5. Cerebrolysin

Cerebrolysin is a low-molecular-weight neuropeptide compound with free amino acids that is derived from the porcine brain tissue. Animal experiments have shown that cerbrolysin has anti-inflammatory properties that can be beneficial in cerebral ischemic stroke. By inducing the expression of anti-inflammatory factors and promoting microglia polarization towards an anti-inflammatory type, cerebrolysin was found to reduce the ischemic infarct volume [79]. Randomized placebo-controlled clinical studies investigating the effect of cerebrolysin in acute ischemic stroke have shown improved 3-month functional outcomes regarding the mRS [80,81,82,83,84,85]. A 10-year retrospective investigation of cerebrolysin treatment in severe aSAH patients found an increase in the survival rates at the 3-month mark for individuals who underwent surgical intervention for aSAH [86]. Woo et al. [38] provided the first results on cerebrolysin in aSAH patients from a prospective randomized double-blind trial. Cerebrolysin appeared to be a safe drug, and mortality was reduced in the cerebrolysin group compared to the placebo group. However, the study size did not allow for this conclusion, and the results regarding functional outcome were also neutral. However, future trials will need to explore cerebrolysin in a larger cohort and with an earlier intervention time-window because significant improvements in 3-month outcomes were found in trials investigating cerebrolysin in ischemic stroke when it was administered within 6 h after hospital admission [82,86].

### 4.6. Dapsone

Dapsone, also known as 4,4′-diamino-diphenylsulfone, is a neuroprotective compound that acts by suppressing glutamate-mediated excitotoxicity and possesses anti-inflammatory properties [87,88]. The anti-inflammatory properties of dapsone are attributed to its ability to hinder the calcium-dependent actions of neutrophils, which includes the suppression of harmful oxidants and proteases released by these cells, leading to tissue damage. Furthermore, dapsone irreversibly inhibits myeloperoxidase, blocks peroxidase functioning in eosinophils, inhibits the synthesis of products by 5-lipoxygenase products, and decreases the levels of neurotoxic free radicals [87,88,89,90]. A prospective randomized and double-blind trial investigating dapsone in acute ischemic stroke revealed that patients receiving dapsone had significant improved neurological functioning at 2 months after acute ischemic stroke compared to the placebo group [90]. To date, only one trial has analyzed the use of dapsone in aSAH patients [27]. Our meta-analysis revealed that this study showed the most promising effect in terms of the probability of a favorable outcome, as defined by the mRS. This beneficial effect appears to be mainly due to the protective role of dapsone in reducing the incidence of vasospasms and DCI. In the placebo group, DCI was present in 63.6% of the patients, while only 26.9% of the patients in the dapsone group had DCI [27]. Future multicenter trials will be necessary to confirm these findings and could also focus on physiological investigations of cerebral blood flow.

### 4.7. Corticosteroids

Corticosteroids are frequently used anti-inflammatory drugs in neurosurgical pathologies due to their diverse functioning as mineralocorticoid or anti-inflammatory drugs. However, there is currently no evidence or guideline supporting the use of corticosteroids to improve neurological outcomes after aSAH [91]. Guidelines suggest maintaining euvolemia in patients with symptomatic delayed cerebral ischemia to reduce the progression and severity of delayed cerebral ischemia, supported by class I evidence [3,4,92,93,94]. Early administration of fludrocortisone was shown to decrease natriuretic diuresis, maintain euvolemia, and reduce symptomatic vasospasm in a study by Nakagawa et al. [95]. Vasospasm is associated with the inflammatory response after aSAH [96], as shown by prospectively recorded serum interleukin-6 levels in 80 patients that were associated with higher Hunt & Hess grades, development of seizure, cerebral vasospasms, and chronic hydrocephalus [97]. Therefore, corticosteroid therapy is assumed to decrease vasospasm by attenuating the inflammatory response. Four investigations analyzed the impact of corticosteroids with minimal mineralocorticoid functions: two trials analyzed methylprednisolone [29,98], whereas two trials utilized dexamethasone [99,100]. Although one randomized controlled trial by Gomis et al. [29] showed a significant reduction of symptomatic vasospasm in high-grade aSAH patients, this effect did not result in a significant improvement of functional outcomes compared to the placebo group. However, the lower boundary of the 95% CI was 0.98 in our pooled analysis, suggesting at least a tendency towards the administration of corticosteroids. Schürkämper et al. [100] investigated dexamethasone in a retrospective study of 242 aSAH patients and found that high-dose (>12 mg/d or >5 days) dexamethasone treatment resulted in an increased probability of favorable outcome compared to those receiving a lower total dose (<12 mg/d or <5 days). However, these results are limited by the retrospective design, and the control group received low amounts of dexamethasone. The ongoing FINISHER trial (**F**ight **IN**flammation to **I**mprove Outcome after Aneurysmal **S**ubarachnoid **HE**mor**R**hage, Trial-Number: NCT05132920) will provide the first data from a prospective randomized placebo-controlled trial evaluating dexamethasone. The primary endpoint of this multicentric trial is the mRS at 6 months after aSAH [101]. This study may elucidate whether the potential benefit of corticosteroids is based on the anti-inflammatory functioning. Furthermore, our current knowledge is based on a combination of several inflammatory pathways that contribute to the inflammatory response after aSAH. Dexamethasone may be an option to simultaneously inhibit several pathways (see Supplementary Figure S2), as the inhibition of single pathways has not shown a significant effect thus far.

This meta-analysis suggests a potential benefit of dapsone or corticosteroids in the management of aSAH, but the conclusions are limited by the low quality of evidence. Furthermore, the present meta-analysis of anti-inflammatory drugs is also limited by the fact that those drugs have different targets and effects. Hence, it is difficult to convex generalizability from the results of each individual study. Nevertheless, even the recent AHA/ASA 2023 aSAH guideline also points out that the emerging data suggesting inflammation to contribute to brain injury in aSAH necessitates further study on glucocorticoid steroids because their safety and efficacy profile is not sufficiently investigated so far [4]. Future studies will have to focus on whether there are different subgroups of aSAH patients who might benefit from different anti-inflammatory drugs targeting individual pathways. Additionally, the findings of the present meta-analysis investigating anti-inflammatory drugs must be interpreted with caution due to different pharmacodynamics and pharmacological effects. Several of the included anti-inflammatory drugs have also other functions, which are paramount regarding the care for aSAH patients. For instance, COX-1 inhibitors have also fundamental effects regarding the inhibition of platelet aggregation, whereas corticosteroids also act on the hemostatic system by increasing clotting factor levels and platelet counts [102,103,104]. The present analysis includes small studies with low statistical power and significant findings, while there are no studies with low power and non-significant results for mortality. Therefore, there may be a publication bias. A major limitation of the evaluation of the neurological outcome are the heterogeneous timepoints of the endpoints of the included studies and the different grades of clinical severity of aSAH, which confound our results. Ultimately, more rigorous data from large, prospective, randomized controlled trials are needed to evaluate the efficacy of dapsone or dexamethasone in the management of aSAH.

## 5. Conclusions

There was no difference in mortality between conventional therapy and anti-inflammatory therapy in patients with aneurysmal subarachnoid hemorrhage. However, certain individual anti-inflammatory therapies, such as dapsone and corticosteroids, may have a positive impact on functional outcomes. To establish sufficient evidence regarding the efficacy of anti-inflammatory therapy in aSAH, further multicenter, randomized, double-blind, and placebo-controlled trials are needed.

## Figures and Tables

**Table 1:** Major characteristics of anti-inflammatory studies included in the present meta-analysis.

Name	Year	Treatments and Sample Size	Doses	Treatment Duration	Recruiting Area
Chou et al. [25]	2008	Simvastatin = 19 versus SNT = 20	80 mg oral daily	Until discharge from NICU or until 21 days	United States of America
Diringer et al. [26]	2016	Simvastatin = 12 versus SNT = 12	80 mg oral daily	21 days	United States of America
Garcia-Pastor et al. [27]	2022	Dapsone = 26 versus SNT = 22	100 mg oral daily	Within 5 days after ictus until day 15	Mexico
Garg et al. [28]	2013	Simvastatin = 19 versus SNT = 19	80 mg daily for 14 days	14 days	India
Gomis et al. [29]	2010	Methylprednisolone = 49 versus SNT = 46	16 mg/kg intravenous daily	3 days	France
Hop et al. [30]	2000	ASA = 24 versus SNT = 26	100 mg suppositories daily	21 days	Netherlands
Katayama et al. [31]	2007	Hydrocortisone = 35 versus SNT = 36	1200 mg/d (day 0–10), 600 mg/d (day 11–12), 300 mg/d (day 13–14) intravenously	14 days	Japan
Kirkpatrick et al. [32]	2014	Simvastatin = 391 versus SNT = 412	40 mg oral daily	3 weeks	UK, Canada, Colombia, Italy, Russia, Singapore, Sweden, Uruguay, USA
Martini et al. [33]	2022	Epoxide hydrolase inhibitor = 10 versus SNT = 10	10 mg oral daily	10 days	United States of America
Suzuki et al. [34]	1989	OKY-046 = 172 versus SNT = 86	80 mg/day (low-dose group) 400 mg/day (high-dose group) intravenously	10 to 14 days	Japan
Tseng et al. [35]	2005	Pravastatin = 40 versus SNT = 40	40 mg oral daily	Within 3 days after ictus for 14 days or until discharge	United Kingdom
Van den Bergh et al. [36]	2006	ASA = 83 versus SNT = 70	100 mg suppositories daily	14 days	Netherlands
Vergouwen et al. [37]	2009	Simvastatin = 14 versus SNT = 16	80 mg oral daily	Within 3 days after SAH until day 14	Netherlands
Woo et al. [38]	2020	Cerebrolysin = 25 versus SNT = 25	30 mL daily for 14 days	14 days	Hong Kong

## Data Availability

All data are included in this manuscript.

## Supplementary Materials

The following supporting information can be downloaded at: https://www.mdpi.com/article/10.3390/jcm12124165/s1, Table S1: Characteristics of excluded anti-inflammatory studies; Table S2: Quality assessment of prospective randomized double-blind, and placebo-controlled trials; Figure S1: PRISMA checklist summarizing the checklist items and the location of the items in the present meta-analysis [105], Figure S2: Illustrative summary of the potential anti-inflammatory functioning and avenues influenced by corticosteroid therapy in aneurysmal subarachnoid hemorrhage.
