# Supplementary material for: Anti-Inflammatory Drug Therapy in Aneurysmal Subarachnoid Hemorrhage: A Systematic Review and Meta-Analysis of Prospective Randomized and Placebo-Controlled Trials
Source: J Clin Med. 2023 Jun 20;12(12):4165. doi: 10.3390/jcm12124165 (PMC10298889; doi:10.3390/jcm12124165)
Supplement: Supplementary file 1 [file jcm-12-04165-s001.zip › jcm-2394120-supplementary.pdf]

SUPPLEMENTARY TABLE S1. Characteristics of excluded anti-inflammatory studies							
Name	Year	Blinding	Treatment	No. patients total	No. patients in treatment	No. patients in control	Cause of exclusion
Chen et al. [39]	2020	Double-blinded	20 mg Atorvastatin for up to 14 days	300	150	150	Only elderly patients (60-90 years)
Fei et al. [40]	2007	Not stated	Intraoperative topical 10 mg dexamethasone	20	10	10	Pilot study, Unknown blinding method No data regarding mortality or mRS follow-up
Galea et al. [41]	2018	Single-blinded	100 mg Subcutaneous interleukin-1 receptor antagonist	136	68	68	Single-blinded
Hasan et al. [42]	1989	Blinded outcome assessment	Intravenous Fludrocortisone acetate or orally 400 µg/d in two doses for 12 days	91	46	45	No placebo, No data regarding mortality or mRS follow-up
Hashi et al. [43]	1988	Double-blinded	Intravenous Hydrocortisone sodium phosphate (3 g) in intervals of 12 hours for 6 times	140	71	69	Written in Japanese
Jaschinski et al. [44]	2008	Not stated	40 mg pravastatin daily	98	40	58	Abstract only, unknown blinding
Lynch et al. [45]	2005	Double-blinded	80 mg Simvastatin daily for 14 days	39	19	20	No data regarding mortality or mRS follow-up
Macedo et al. [46]	2009	Not blinded	80 mg simvastatin	21	11	10	Abstract only, not blinded
Mori et al. [47]	1999	Not stated	Oral Fludrocortisone acetate 0.3 mg/d for 1-8 days	25	15	10	Unknown Blinding method, unknown randomization method, no placebo
Moro et al. [48]	2003	Not blinded	1200 mg Hydrocortisone per day until day 10	28	14	14	Not blinded, no placebo, no data regarding mortality or mRS follow-up
Naraoka et al. [49]	2018	Double-blinded	4 mg Pitavastatin daily for 14 days	108	54	54	No data regarding mortality or mRS follow-up
Singh et al. [50]	2014	Double-blinded	Intravenous bolus of IL-1 Receptor Antagonist	13	6	7	No data regarding mortality or mRS follow-up
Wong et al. [51]	2014	Double-blinded	80 or 40 mg Simvastatin daily for 21 days	255	131 (high dose)	124 (low dose)	No placebo

**SUPPLEMENTARY TABLE S2**: Quality assessment of prospective randomized double-blind, and placebo-controlled trials		
**Chou 2008 [25]**	**Risk of bias**	**Author judgment**
Random sequence generation (selection bias)	Unclear	Randomization was performed. Randomization sequence is not described.
Allocation concealment (selection bias)	Unclear	The allocation concealment is not reported.
Blinding of participants and personnel (performance bias)	Low risk	Double blinded
Blinding of outcome assessment (detection bias)	Low risk	Double blinded
Incomplete outcome data (attrition bias)	Low risk	All outcome data are reported. Intention-to-treat analyses were performed
Selective reporting (reporting bias)	Low risk	All prespecified outcomes were reported.
Other bias	Unclear	
**Diringer 2016 [26]**	**Risk of bias**	**Author judgment**
Random sequence generation (selection bias)	Unclear	Randomization was performed by a computerized system. Randomization sequence is not described.
Allocation concealment (selection bias)	Unclear	The allocation concealment is not reported.
Blinding of participants and personnel (performance bias)	Low risk	Double blinded
Blinding of outcome assessment (detection bias)	Low risk	Double blinded
Incomplete outcome data (attrition bias)	Low risk	All outcome data are reported.
Selective reporting (reporting bias)	Low risk	All prespecified outcomes were reported.
Other bias	Unclear	
**Garcia Pastor 2022 [27]**	**Risk of bias**	**Author judgment**
Random sequence generation (selection bias)	Unclear	Computer-derived tables were used for randomization. Further information is not given
Allocation concealment (selection bias)	Unclear	Patients were randomly allocated. Detailed allocation concealment not given.
Blinding of participants and personnel (performance bias)	Low risk	Double blinded
Blinding of outcome assessment (detection bias)	Low risk	Double blinded
Incomplete outcome data (attrition bias)	Unclear	All prespecified endpoints are summarized with the results. Incomplete outcome data of mRS score at 3 months. Reason for lack of data in a few patients not available.
Selective reporting (reporting bias)	Low risk	All prespecified outcomes were reported.
Other bias	Unclear	
**Garg 2013 [28]**	**Risk of bias**	**Author judgment**
Random sequence generation (selection bias)	Low risk	Randomization was performed. Patients were randomized by block randomization method with variable block size.
Allocation concealment (selection bias)	Low risk	Allocation to each group in a 1:1 ratio.
Blinding of participants and personnel (performance bias)	Low risk	Double blinded
Blinding of outcome assessment (detection bias)	Low risk	Double blinded
Incomplete outcome data (attrition bias)	Low risk	All outcome data are reported. Drug discontinuation because of transaminitis in both arms.
Selective reporting (reporting bias)	Low risk	All prespecified outcomes were reported.
Other bias	Unclear	

Gomis 2010 [29]	Risk of bias	Author judgment
Random sequence generation (selection bias)	Unclear	The method of randomization is not reported.
Allocation concealment (selection bias)	Unclear	The allocation concealment is not reported.
Blinding of participants and personnel (performance bias)	Low risk	Double blinded
Blinding of outcome assessment (detection bias)	Low risk	Double blinded
Incomplete outcome data (attrition bias)	Low risk	One patient of the experimental arm and three of the placebo arm were regarded as not treatable because of early death by recurrent hemorrhages prior to treatment, early death after first hemorrhage, acute renal failure, acute respiratory failure, and hypoglycemia. Further outcome data are present in all patients
Selective reporting (reporting bias)	Low risk	All prespecified outcomes were reported.
Other bias	Unclear	
**Hop 2000 [30]**	**Risk of bias**	**Author judgment**
Random sequence generation (selection bias)	Unclear	The method of randomization is not reported.
Allocation concealment (selection bias)	Unclear	The allocation concealment is not reported.
Blinding of participants and personnel (performance bias)	Low risk	Double blinded
Blinding of outcome assessment (detection bias)	Low risk	Double blinded
Incomplete outcome data (attrition bias)	Low risk	All outcome data are reported.
Selective reporting (reporting bias)	Low risk	All prespecified outcomes were reported.
Other bias	Unclear	
**Katayama 2007 [31]**	**Risk of bias**	**Author judgment**
Random sequence generation (selection bias)	Unclear	Randomization was performed. Randomization sequence is not reported.
Allocation concealment (selection bias)	Unclear	Allocation concealment is not reported.
Blinding of participants and personnel (performance bias)	Low risk	Double blinded
Blinding of outcome assessment (detection bias)	Low risk	Double blinded
Incomplete outcome data (attrition bias)	Low risk	All outcome data are reported.
Selective reporting (reporting bias)	Low risk	All prespecified outcomes were reported.
Other bias	Unclear	
**Kirkpatrick 2014 [32]**	**Risk of bias**	**Author judgment**
Random sequence generation (selection bias)	Low risk	Randomization was performed. Randomization sequence was generated by a computer to randomize patients by blocks of ten patients (5 experiment, 5 placebo).
Allocation concealment (selection bias)	Low risk	Allocation to each group in a 1:1 ratio.
Blinding of participants and personnel (performance bias)	Low risk	Double blinded
Blinding of outcome assessment (detection bias)	Low risk	Double blinded
Incomplete outcome data (attrition bias)	Low risk	All outcome data are reported. Lack of data and exclusion of patients are explained. In both arms patients withdrew, were not adherent to drug protocol, and were lost to

		follow-up. Intention-to-treat analyses were performed
Selective reporting (reporting bias)	Low risk	All prespecified outcomes were reported.
Other bias	Unclear	
**Martini 2022 [33]**	**Risk of bias**	**Author judgment**
Random sequence generation (selection bias)	Low risk	The method of randomization is reported. Computer-derived random sequence based on an unrestricted "fair-coin" randomization procedure was provided by an independent statistician.
Allocation concealment (selection bias)	Low risk	Allocation to each group in a 1:1 ratio.
Blinding of participants and personnel (performance bias)	Low risk	Double blinded
Blinding of outcome assessment (detection bias)	Low risk	Double blinded
Incomplete outcome data (attrition bias)	Low risk	One patient of the placebo group withdrew consent prior to receiving the first dose. Further outcome data are present.
Selective reporting (reporting bias)	Low risk	All prespecified outcomes were reported.
Other bias	Unclear	
**Suzuki 1989 [34]**	**Risk of bias**	**Author judgment**
Random sequence generation (selection bias)	Unclear	Randomization was performed. Randomization sequence is not reported.
Allocation concealment (selection bias)	Unclear	Allocation concealment is not reported.
Blinding of participants and personnel (performance bias)	Low risk	Double blinded
Blinding of outcome assessment (detection bias)	Low risk	Double blinded
Incomplete outcome data (attrition bias)	Low risk	All outcome data are reported.
Selective reporting (reporting bias)	Low risk	All prespecified outcomes were reported.
Other bias	Unclear	
**Tseng 2005 [35]**	**Risk of bias**	**Author judgment**
Random sequence generation (selection bias)	Low risk	Simple randomization was used
Allocation concealment (selection bias)	Unclear	The allocation concealment is not reported.
Blinding of participants and personnel (performance bias)	Low risk	Double blinded
Blinding of outcome assessment (detection bias)	Low risk	Double blinded
Incomplete outcome data (attrition bias)	Low risk	All outcome data are reported.
Selective reporting (reporting bias)	Low risk	All prespecified outcomes were reported.
Other bias	Unclear	
**Van den Bergh 2006 [36]**	**Risk of bias**	**Author judgment**
Random sequence generation (selection bias)	Low-risk	The method of randomization is reported. Factorial design was used
Allocation concealment (selection bias)	Low-risk	Allocation to each group in a 1:1 ratio.
Blinding of participants and personnel (performance bias)	Low-risk	Double blinded
Blinding of outcome assessment (detection bias)	Low-risk	Double blinded
Incomplete outcome data (attrition bias)	Low risk	Discontinued intervention in both arms because of adverse events. Intention-to-treat analysis was performed
Selective reporting (reporting bias)	Low risk	All prespecified outcomes were reported
Other bias	Unclear	
**Vergouwen 2009 [37]**	**Risk of bias**	**Author judgment**

Random sequence generation (selection bias)	Low risk	Randomization was performed. Permuted-block randomization was used.
Allocation concealment (selection bias)	Low risk	Allocation to each group in a 1:1 ratio.
Blinding of participants and personnel (performance bias)	Low risk	Double blinded
Blinding of outcome assessment (detection bias)	Low risk	Double blinded
Incomplete outcome data (attrition bias)	Low risk	All outcome data are reported except for those patients with a discontinued intervention because of adverse events or study medication lost. Intention-to-treat analyses were performed
Selective reporting (reporting bias)	Low risk	All prespecified outcomes were reported.
Other bias	Unclear	
**Woo 2020 [38]**	**Risk of bias**	**Author judgment**
Random sequence generation (selection bias)	Low risk	Randomization was performed. Randomization sequence was generated by SPSS with a block size of ten patients.
Allocation concealment (selection bias)	Low risk	Allocation to each group in a 1:1 ratio.
Blinding of participants and personnel (performance bias)	Low risk	Double blinded
Blinding of outcome assessment (detection bias)	Low risk	Double blinded
Incomplete outcome data (attrition bias)	Low risk	All outcome data are reported. Analyses were performed on a modified intention-to-treat basis.
Selective reporting (reporting bias)	Low risk	All prespecified outcomes were reported.
Other bias	Unclear	

PRISMA 2020 Checklist

Section and Topic	Item #	Checklist item	Location where item is reported
TITLE			
Title	1	Anti-inflammatory drug therapy in aneurysmal subarachnoid hemorrhage: A systematic review and meta-analysis of prospective randomized and placebo-controlled trials	Title
ABSTRACT			
Abstract	2	Emerging evidence suggests a potential role of neuroinflammation in aneurysmatic subarachnoid hemorrhage (aSAH). We aim to investigate the impact of anti-inflammatory therapy on survival and outcome in aSAH. Eligible randomized placebo-controlled prospective trials (RCT) were searched in PubMed in February 2023. After the available studies following inclusion and exclusion criteria were screened, the main outcome measures were strictly extracted.	Abstract
INTRODUCTION			
Rationale	3	Delayed cerebral ischemia (DCI) is associated with cerebral vasospasm (CVS) and is suggested to be related to the poor outcome after aSAH. To improve outcome, rescue therapies including selective intraarterial infusion of vasodilators, balloon angioplasty, as well as induced hypertension are used. However, the underlying pathophysiological mechanism enabling the injury expansion following aSAH is still not completely understood. Hence, there is still a limited number of potential proven effective pharmacological drug therapy options. Emerging evidence suggests neuroinflammation as a main avenue of injury expansion and neurological deficits. The peripheral immune cells are recruited into the brain parenchyma and their activated forms release inflammatory cytokines. Vessels being affected by cerebral vasospasm have been found to have an elevated leukocyte adhesion capacity which results in delayed neurologic deterioration.	Introduction
Objectives	4	In the present meta-analysis, we aim to investigate the existing evidence and identify effective drug interventions that might improve functional outcome and survival rates in patients with aSAH.	Introduction
METHODS			
Eligibility criteria	5	The authors conducted a systematic search of the Pubmed database (http://www.ncbi.nlm.nih.gov/pubmed) in July 2022 for the terms "aneurysmal subarachnoid hemorrhage", "SAH", and "subarachnoid hemorrhage". The search was limited to "randomized controlled trials", "human studies" and "English". The articles were screened for randomized placebo, controlled trials investigating anti-inflammatory drug therapies. The literature search included all results until 30 June 2022. Inclusion criteria were formulated according to the PICOS (population, intervention, comparator, outcomes and study design) framework [19]. These criteria were defined as follows: patients suffered from an aSAH; anti-inflammatory drug therapies were conducted; results were compared to a placebo arm; all results of the prespecified endpoints were reported; and the trials were defined as prospective, randomized, placebo-controlled, and double-blinded studies. The following types of results were excluded: reviews, letters, study protocols, conference abstracts, unpublished manuscripts, animal experiments, and studies with insufficient data (e.g., randomized controlled trials without a placebo arm). Furthermore, previous meta-analyses and reviews were searched for studies matching our inclusion and exclusion criteria. The identified articles were evaluated for relevance in a step-wise workflow: First the study titles were screened, then the corresponding abstract and in case of further uncertainty the full-text was screened by two authors (JW and EG) until all retrieved studies were either included or excluded.	Methods
Information sources	6	The authors conducted a systematic search of the Pubmed database (http://www.ncbi.nlm.nih.gov/pubmed) in July 2022 for the terms "aneurysmal subarachnoid hemorrhage", "SAH", and "subarachnoid hemorrhage".	Methods
Search strategy	7	The search was limited to "randomized controlled trials", "human studies" and "English". The articles were screened for randomized placebo,	Methods

Section and Topic	Item #	Checklist item	Location where item is reported
		controlled trials investigating anti-inflammatory drug therapies. The literature search included all results until 31 March 2023.	
Selection process	8	We performed title screening, abstract screening and in case of uncertainty a whole text screening to search for the eligible studies.	Methods
Data collection process	9	The data collection was performed by two authors independently (MV, JW). First the study titles were screened, then the corresponding abstract and in case of further uncertainty the full-text was screened by two authors (JW and EG) until all retrieved studies were either included or excluded.	Methods
Data items	10a	We analyzed the studies to conduct a meta-analysis according to the following outcomes:<br>1) In case of administration of anti-inflammatory drugs, following outcomes were analyzed: Mortality, Outcome	Methods
	10b	Mortality was assessed according to provided data as patients, who deceased in the course of the therapy<br>We defined general outcome according to modified Ranking scale (mRS) and dichotomized into good (mRS 0-2) and poor (mRS 3-5)	Methods
Study risk of bias assessment	11	The Cochrane Bias Risk Tool was used to analyze the risk of bias (ROB) in the included studies using the software Review Manager Web (RevMan Web Version 5.4.1 from The Cochrane Collaboration, available at revman.cochrane.org (accessed on 21 December 2022). The following six characteristics regarding risk of bias assessment were included in the analysis: selection bias, performance bias, detection bias, attrition bias, reporting bias, and other sources of bias. Afterwards, a risk of bias summary chart and plot were created. Two reviewers screened the records independently.	Methods
Effect measures	12	We measured and reported outcomes in Forest-plots, providing heterogeneity and inconsistency analysis, pooled odds ratios and statistical significance	Methods
Synthesis methods	13a	To see all the eligible studies and reported outcomes, see Table 1	Methods
	13b	To see all the eligible studies and reported outcomes, see Table 1	Methods
	13c	For visualization of our meta-analysis, Forest Plots were created with Review Manager Web (RevMan Web Version 5.4.1 from the Cochrane Collaboration, available at revman.cochrane.org) were presented as figures to selected outcomes	Methods
	13d	Review Manger Web (RevMan Web Version 5.4.1 from The Cochrane Collaboration) was used to conduct the meta-analyses. To analyze the statistical heterogeneity and inconsistency, x^2 and I^2 statistics were used, respectively; an I^2 value of 50% or more represented substantial heterogeneity. Weight of the relative contribution of the individual trials, based on study sample size, was considered regarding the treatment effect estimation. Data from a multi-arm trial with different dosage regimens were extracted by combining those groups for the inclusion in the pairwise meta-analysis [21]. Publication bias of the included studies regarding both defined endpoints (survival & neurological outcome) was assessed using three different methods: (1) Funnel plots were created to visually investigate the publication bias of the included trials; (2) An egger regression was performed to statistically evaluate the symmetry of the funnel plot. The likelihood of publication bias was determined using the Egger regression intercept two-tailed test and the threshold of the significance value was set at 5% [22,]. (3) Begg´s test was used for the evaluation of the data symmetry [23]. Egger´s and Begg´s tests were performed using MedCalc (Version 20.123 for Windows). Effect sizes were expressed as pooled odds ratio (OR) estimates using a random effect model. Analyses for both death and poor outcome were performed	Methods
	13e	In case of subgroup-analysis, there might be a heterogeneity of the data caused by lack of published results	Methods
	13f	Not available	Methods

PRISMA 2020 Checklist

Section and Topic	Item #	Checklist item	Location where item is reported
Reporting bias assessment	14	According to our strict inclusion criteria and high quality of included studies, we do not suppose to have bias due to missing results. Risk of bias assessment was performed for all included studies	Methods
Certainty assessment	15	Quality assessment was performed and the risk of bias assessment is summarized in supplementary table S2.	Methods
RESULTS			
Study selection	16a	Process of the search and selection is summarized in Figure 1.	Figure 1
	16b	Excluded studies are summarized in supplementary Table S1	Table S1
Study characteristics	17	Included studies are summarized in table 1.	Table 1
Risk of bias in studies	18	Risk of bias analysis is reported in supplementary table 2 and Figure 2	Table S2 & Figure 2
Results of individual studies	19	Analysis of each outcome is separately reported on with it´s own forest plot	Figures 2-4
Results of syntheses	20a	Risk of bias assessment is summarized in the Figure 2. Figure 4 represent funnel plots visualizing the publication bias	Figure 2 & 4
	20b	Results of the statistical syntheses are summarized in the sections 3.4-3.6	Results 3.4-3.6
	20c	Results regarding heterogeneity are presented in the sections 3.4-3.6	Results 3.4-3.6
	20d	Presented	Results 3.4-3.6
Reporting biases	21	Risk of bias assessment is shown in section 3.3	Results 3.3
Certainty of evidence	22	Not available	NA
DISCUSSION			
Discussion	23a	Provide a general interpretation of the results in the context of other evidence.<br>Presented	Discussion
	23b	Discuss any limitations of the evidence included in the review.<br>Presented	Discussion, last paragraph
	23c	Discuss any limitations of the review processes used.<br>Presented	Discussion
	23d	Discuss implications of the results for practice, policy, and future research.	Discussion

PRISMA 2020 Checklist

Section and Topic	Item #	Checklist item	Location where item is reported
		Presented	
OTHER INFORMATION			
Registration and protocol	24a	The study was registered in the "International Prospective Register of Systematic Reviews" (PROSPERO) in 2023 (CRD42023395375)	
	24b	The study was registered in the "International Prospective Register of Systematic Reviews" (PROSPERO) in 2023 (CRD42023395375)	
	24c	No amendments to describe	
Support	25	There is no financial conflict of interest to declare	
Competing interests	26	No competing interests of the authors to delcare	
Availability of data, code and other materials	27	Template data collection form, data extracted and used for the analysis are reported in the section "methods". The softwares Revman and Medcalc are available online	

From: Page MJ, McKenzie JE, Bossuyt PM, Boutron I, Hoffmann TC, Mulrow CD, et al. The PRISMA 2020 statement: an updated guideline for reporting systematic reviews. BMJ 2021;372:n71. doi: 10.1136/bmj.n71

For more information, visit: http://www.prisma-statement.org/

Figure S1. PRISMA checklist summarizing the checklist items and the location of the items in the present meta-analysis.

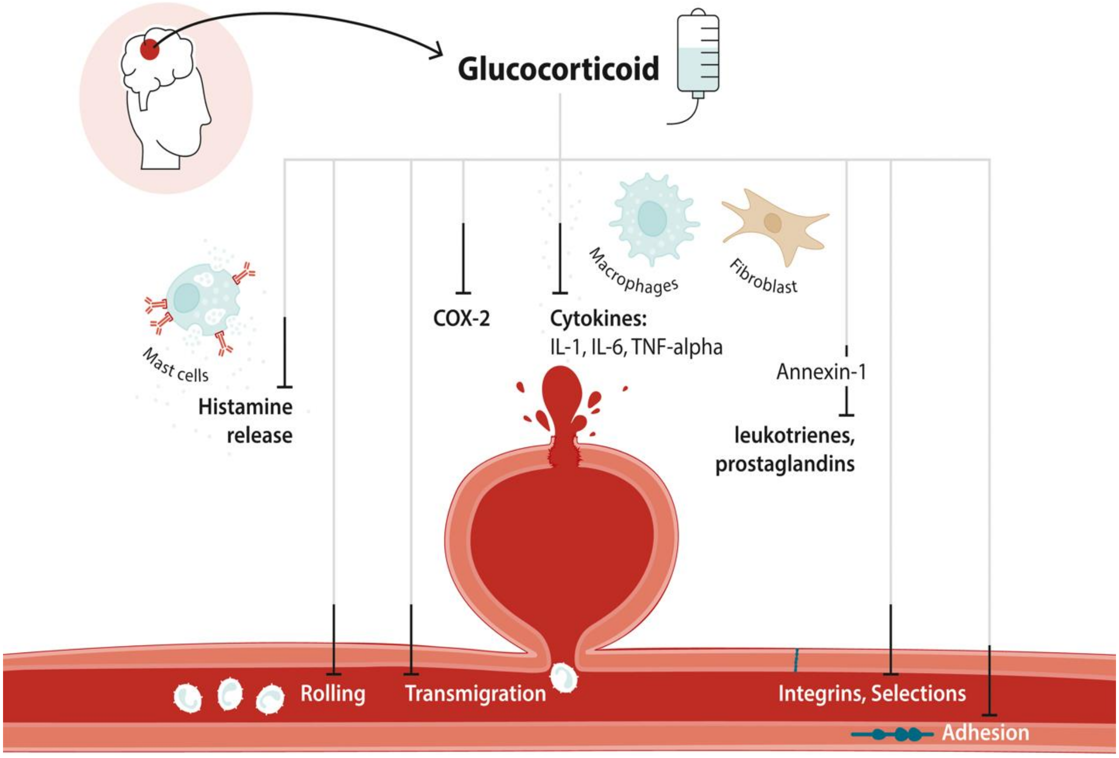

Figure S2. Illustrative summary of the potential anti-inflammatory functioning and avenues influenced by corticosteroid therapy in aneurysmal subarachnoid hemorrhage.
